# Perhydro­benzimidazole-2-thione

**DOI:** 10.1107/S1600536808043894

**Published:** 2009-01-08

**Authors:** YingChun Liu, XiaoYu Li

**Affiliations:** a Department of Biomedicine, Zhongshan Torch Polytechnic, Zhongshan 528436, Guangdong Province, People’s Republic of China

## Abstract

The studied crystal of the title compound, C_7_H_12_N_2_S, is a racemic mixture of two isomers, *viz. S*,*S* and *R*,*R*. The two isomers share the same position on a mirror plane in the space group *P*2_1_/*m*; thus all atoms except one are disordered between two positions in a 1:1 ratio. Inter­molecular N—H⋯S hydrogen bonds link the mol­ecules into chains propagating in the [010] direction.

## Related literature

For details of the synthesis, see: Allen *et al.* (1946 ▶). For useful applications of thio­urea derivetives, see: Schroeder (2006 ▶); Amos *et al.* (2007 ▶).
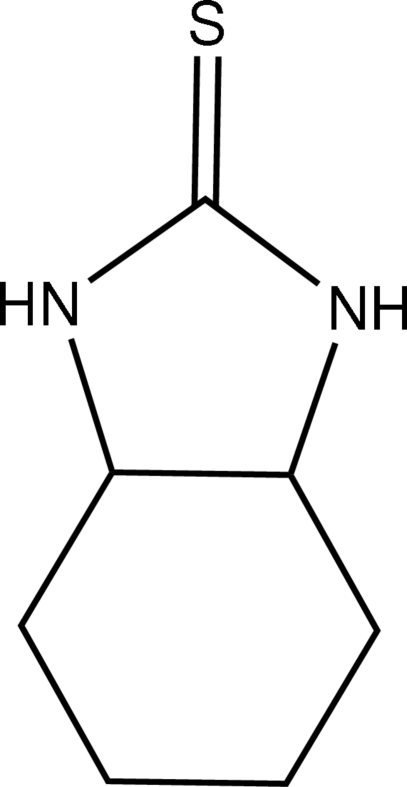


## Experimental

### 

#### Crystal data

C_7_H_12_N_2_S
*M* *_r_* = 156.25
Monoclinic, 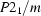

*a* = 5.7459 (16) Å
*b* = 8.543 (2) Å
*c* = 8.816 (2) Å
β = 98.208 (4)°
*V* = 428.3 (2) Å^3^
*Z* = 2
Mo *K*α radiation
μ = 0.31 mm^−1^
*T* = 293 (2) K
0.20 × 0.10 × 0.10 mm

#### Data collection

Bruker SMART CCD area-detector diffractometer
Absorption correction: multi-scan (*SADABS*; Sheldrick, 1996 ▶) *T*_min_ = 0.931, *T*_max_ = 0.970
4541 measured reflections
934 independent reflections
740 reflections with *I* > 2σ(*I*)
*R*_int_ = 0.019

#### Refinement

*R*[*F* ^2^ > 2σ(*F* ^2^)] = 0.047
*wR*(*F* ^2^) = 0.154
*S* = 1.03
934 reflections
91 parameters
6 restraints
H-atom parameters constrained
Δρ_max_ = 0.19 e Å^−3^
Δρ_min_ = −0.14 e Å^−3^

### 

Data collection: *SMART* (Bruker, 1997 ▶); cell refinement: *SAINT* (Bruker, 1999 ▶); data reduction: *SAINT*; program(s) used to solve structure: *SHELXS97* (Sheldrick, 2008 ▶); program(s) used to refine structure: *SHELXL97* (Sheldrick, 2008 ▶); molecular graphics: *SHELXTL* (Sheldrick, 2008 ▶); software used to prepare material for publication: *SHELXTL*.

## Supplementary Material

Crystal structure: contains datablocks I, global. DOI: 10.1107/S1600536808043894/cv2499sup1.cif

Structure factors: contains datablocks I. DOI: 10.1107/S1600536808043894/cv2499Isup2.hkl

Additional supplementary materials:  crystallographic information; 3D view; checkCIF report

## Figures and Tables

**Table 1:** Hydrogen-bond geometry (Å, °)

*D*—H⋯*A*	*D*—H	H⋯*A*	*D*⋯*A*	*D*—H⋯*A*
N1*A*—H1*A*⋯S1*A*^i^	0.86	2.53	3.367 (11)	166
N1*B*—H1*B*⋯S1*B*^ii^	0.86	2.76	3.483 (11)	142

Symmetry codes: (i) 
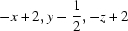
; (ii) 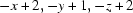.

**Fig. 1.**
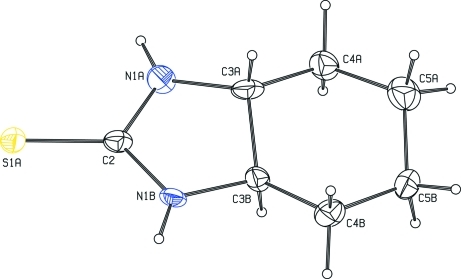
View (S,S)-isomer of (I) showing the atom-labelling scheme. Displacement ellipsoids are drawn at the 30% probability level.

## supplementary crystallographic information

### Comment

Thiourea and its derivatives are used in dyes, photographic film, elastomers, plastics, textiles, insecticides, preservatives, rodenticides and pharmaceuticals (Schroeder *et al.*, 2006; Amos *et al.*, 2007)

The title molecule consists of one thioimidazole five-membered ring and one six-membered ring which display chair conformation. The studied crystal is a racemic mixture of two isomers - (S,S) and (R,R), respectively - which share the same position on a mirror plane in space group P2_1_/m, thus all atoms except one are disordered between two positions in a ratio 1:1. In the crystal, intermolecular N—H···S hydrogen bonds (Table 1) link the molecules into chains propagating in direction [010].

### Experimental

The title compound was prepared according to the reported method (Allen *et al.*,1946). Crystals of (I) suitable for X-ray data collection were obtained by slow evaporation of a CH_2_Cl_2_ and MeOH solution in a ratio of 4:1 at 293 K.

### Refinement

All H atoms were geometrically positioned (C–H 0.97-0.98 Å, N–H 0.86 Å) and refined as riding, with  U_iso_(H) = 1.2 *U*_eq_(C, N). The crystal structure was refined in two space groups - P2_1_ and P2_1_/m, respectively. In both groups the severe disorder has been observed with almost identical values of final R-factors, so the preference has been made for P2_1_/m.

### Crystal data

**Table:**

C_7_H_12_N_2_S	*F*(000) = 168
*M**_r_* = 156.25	*D*_x_ = 1.211 Mg m^−^^3^
Monoclinic, *P*2_1_/*m*	Mo *K*α radiation, λ = 0.71073 Å
Hall symbol: -P 2yb	Cell parameters from 1728 reflections
*a* = 5.7459 (16) Å	θ = 2.3–24.6°
*b* = 8.543 (2) Å	µ = 0.31 mm^−^^1^
*c* = 8.816 (2) Å	*T* = 293 K
β = 98.208 (4)°	Block, colourless
*V* = 428.3 (2) Å^3^	0.20 × 0.10 × 0.10 mm
*Z* = 2	

### Data collection

**Table:**

Bruker SMART CCD area-detector diffractometer	934 independent reflections
Radiation source: fine-focus sealed tube	740 reflections with *I* > 2σ(*I*)
graphite	*R*_int_ = 0.019
φ and ω scans	θ_max_ = 26.5°, θ_min_ = 2.3°
Absorption correction: multi-scan (*SADABS*; Sheldrick, 1996)	*h* = −7→7
*T*_min_ = 0.931, *T*_max_ = 0.970	*k* = −9→10
4541 measured reflections	*l* = −11→11

### Refinement

**Table:**

Refinement on *F*^2^	Primary atom site location: structure-invariant direct methods
Least-squares matrix: full	Secondary atom site location: difference Fourier map
*R*[*F*^2^ > 2σ(*F*^2^)] = 0.047	Hydrogen site location: inferred from neighbouring sites
*wR*(*F*^2^) = 0.154	H-atom parameters constrained
*S* = 1.03	*w* = 1/[σ^2^(*F*_o_^2^) + (0.1091*P*)^2^ + 0.0156*P*] where *P* = (*F*_o_^2^ + 2*F*_c_^2^)/3
934 reflections	(Δ/σ)_max_ = 0.009
91 parameters	Δρ_max_ = 0.19 e Å^−^^3^
6 restraints	Δρ_min_ = −0.14 e Å^−^^3^

### Special details

**Table:**

Geometry. All esds (except the esd in the dihedral angle between two l.s. planes) are estimated using the full covariance matrix. The cell esds are taken into account individually in the estimation of esds in distances, angles and torsion angles; correlations between esds in cell parameters are only used when they are defined by crystal symmetry. An approximate (isotropic) treatment of cell esds is used for estimating esds involving l.s. planes.
Refinement. Refinement of F^2^ against ALL reflections. The weighted R-factor wR and goodness of fit S are based on F^2^, conventional R-factors R are based on F, with F set to zero for negative F^2^. The threshold expression of F^2^ > 2sigma(F^2^) is used only for calculating R-factors(gt) etc. and is not relevant to the choice of reflections for refinement. R-factors based on F^2^ are statistically about twice as large as those based on F, and R- factors based on ALL data will be even larger.

### Fractional atomic coordinates and isotropic or equivalent isotropic displacement parameters (Å^2^)

**Table:**

	*x*	*y*	*z*	*U*_iso_*/*U*_eq_	Occ. (<1)
C2	0.8296 (4)	0.2500	0.9716 (3)	0.0734 (7)	
S1A	1.0495 (14)	0.2500	1.1194 (10)	0.0811 (15)	0.50
N1A	0.746 (3)	0.1176 (10)	0.9007 (16)	0.095 (4)	0.50
H1A	0.8101	0.0266	0.9121	0.113*	0.50
C3A	0.534 (2)	0.1541 (15)	0.8039 (15)	0.102 (4)	0.50
H3A	0.4166	0.1316	0.8715	0.122*	0.50
C4A	0.4237 (9)	0.0818 (6)	0.6596 (6)	0.0974 (14)	0.50
H4A1	0.3843	−0.0258	0.6803	0.117*	0.50
H4A2	0.5382	0.0796	0.5887	0.117*	0.50
C5A	0.2070 (17)	0.1621 (11)	0.5834 (11)	0.119 (6)	0.50
H5A1	0.0758	0.1270	0.6327	0.143*	0.50
H5A2	0.1779	0.1270	0.4777	0.143*	0.50
S1B	1.0773 (15)	0.2500	1.0974 (10)	0.088 (2)	0.50
N1B	0.697 (2)	0.3722 (7)	0.9103 (13)	0.0720 (19)	0.50
H1B	0.7108	0.4663	0.9453	0.086*	0.50
C3B	0.5339 (13)	0.3261 (13)	0.7810 (14)	0.0718 (18)	0.50
H3B	0.6275	0.3463	0.6985	0.086*	0.50
C4B	0.3201 (9)	0.4183 (6)	0.7250 (7)	0.0994 (15)	0.50
H4B1	0.3630	0.5236	0.6986	0.119*	0.50
H4B2	0.2188	0.4249	0.8039	0.119*	0.50
C5B	0.1951 (16)	0.3360 (13)	0.5860 (11)	0.121 (6)	0.50
H5B1	0.0328	0.3707	0.5709	0.146*	0.50
H5B2	0.2648	0.3707	0.4979	0.146*	0.50

### Atomic displacement parameters (Å^2^)

**Table:**

	*U*^11^	*U*^22^	*U*^33^	*U*^12^	*U*^13^	*U*^23^
C2	0.0817 (15)	0.0481 (12)	0.0918 (16)	0.000	0.0170 (12)	0.000
S1A	0.094 (2)	0.0635 (17)	0.0790 (14)	0.000	−0.010 (3)	0.000
N1A	0.079 (6)	0.063 (4)	0.136 (6)	0.015 (2)	−0.001 (4)	−0.013 (3)
C3A	0.141 (8)	0.044 (3)	0.118 (7)	−0.013 (3)	0.008 (5)	0.009 (4)
C4A	0.096 (3)	0.074 (3)	0.119 (4)	0.003 (3)	0.000 (3)	−0.018 (3)
C5A	0.112 (7)	0.091 (8)	0.134 (8)	−0.016 (5)	−0.050 (5)	−0.018 (6)
S1B	0.105 (2)	0.0474 (14)	0.114 (4)	0.000	0.0191 (14)	0.000
N1B	0.077 (5)	0.0334 (19)	0.102 (3)	−0.008 (2)	0.002 (3)	−0.002 (2)
C3B	0.063 (3)	0.052 (3)	0.096 (3)	0.006 (2)	−0.003 (2)	−0.014 (3)
C4B	0.096 (4)	0.070 (3)	0.130 (4)	0.022 (3)	0.009 (3)	0.010 (3)
C5B	0.098 (7)	0.122 (11)	0.148 (9)	−0.009 (5)	0.030 (5)	−0.011 (6)

### Geometric parameters (Å, °)

**Table:**

C2—N1A	1.348 (6)	C5A—C5A^i^	1.502 (19)
C2—N1A^i^	1.348 (6)	C5A—H5A1	0.9700
C2—N1B	1.357 (5)	C5A—H5A2	0.9700
C2—N1B^i^	1.357 (5)	N1B—C3B	1.426 (7)
C2—S1B	1.675 (5)	N1B—H1B	0.8600
C2—S1A	1.680 (4)	C3B—C3B^i^	1.30 (2)
N1A—C3A	1.420 (8)	C3B—C4B	1.483 (7)
N1A—H1A	0.8600	C3B—H3B	0.9800
C3A—C4A	1.473 (8)	C4B—C5B	1.504 (7)
C3A—C3A^i^	1.64 (3)	C4B—H4B1	0.9700
C3A—H3A	0.9800	C4B—H4B2	0.9700
C4A—C5A	1.494 (7)	C5B—C5B^i^	1.47 (2)
C4A—H4A1	0.9700	C5B—H5B1	0.9700
C4A—H4A2	0.9700	C5B—H5B2	0.9700
			
N1A—C2—N1A^i^	114.2 (10)	C4A—C5A—C5A^i^	117.3 (4)
N1A—C2—N1B	108.6 (3)	C4A—C5A—H5A1	108.0
N1A^i^—C2—N1B^i^	108.6 (3)	C5A^i^—C5A—H5A1	108.0
N1B—C2—N1B^i^	100.6 (9)	C4A—C5A—H5A2	108.0
N1A—C2—S1B	121.3 (5)	C5A^i^—C5A—H5A2	108.0
N1A^i^—C2—S1B	121.3 (6)	H5A1—C5A—H5A2	107.2
N1B—C2—S1B	129.6 (4)	C2—N1B—C3B	111.9 (5)
N1B^i^—C2—S1B	129.6 (4)	C2—N1B—H1B	124.0
N1A—C2—S1A	122.6 (5)	C3B—N1B—H1B	124.0
N1A^i^—C2—S1A	122.6 (5)	C3B^i^—C3B—N1B	106.0 (4)
N1B—C2—S1A	128.7 (4)	C3B^i^—C3B—C4B	122.1 (5)
N1B^i^—C2—S1A	128.7 (4)	N1B—C3B—C4B	122.6 (11)
C2—N1A—C3A	108.2 (8)	C3B^i^—C3B—H3B	100.1
C2—N1A—H1A	125.9	N1B—C3B—H3B	100.1
C3A—N1A—H1A	125.9	C4B—C3B—H3B	100.1
N1A—C3A—C4A	130.7 (11)	C3B—C4B—C5B	107.4 (7)
N1A—C3A—C3A^i^	102.7 (5)	C3B—C4B—H4B1	110.2
C4A—C3A—C3A^i^	114.8 (6)	C5B—C4B—H4B1	110.2
N1A—C3A—H3A	101.3	C3B—C4B—H4B2	110.2
C4A—C3A—H3A	101.3	C5B—C4B—H4B2	110.2
C3A^i^—C3A—H3A	101.3	H4B1—C4B—H4B2	108.5
C3A—C4A—C5A	115.0 (7)	C5B^i^—C5B—C4B	117.9 (5)
C3A—C4A—H4A1	108.5	C5B^i^—C5B—H5B1	107.8
C5A—C4A—H4A1	108.5	C4B—C5B—H5B1	107.8
C3A—C4A—H4A2	108.5	C5B^i^—C5B—H5B2	107.8
C5A—C4A—H4A2	108.5	C4B—C5B—H5B2	107.8
H4A1—C4A—H4A2	107.5	H5B1—C5B—H5B2	107.2
			
N1A^i^—C2—N1A—C3A	−21 (2)	N1A—C2—N1B—C3B	−6.9 (9)
N1B—C2—N1A—C3A	−7.6 (10)	N1A^i^—C2—N1B—C3B	110 (5)
N1B^i^—C2—N1A—C3A	47 (3)	N1B^i^—C2—N1B—C3B	−18 (2)
S1B—C2—N1A—C3A	179.0 (10)	S1B—C2—N1B—C3B	165.7 (10)
S1A—C2—N1A—C3A	168.3 (11)	S1A—C2—N1B—C3B	177.5 (9)
C2—N1A—C3A—C4A	151.0 (13)	C2—N1B—C3B—C3B^i^	11.8 (14)
C2—N1A—C3A—C3A^i^	11.4 (13)	C2—N1B—C3B—C4B	159.0 (10)
N1A—C3A—C4A—C5A	−175.2 (15)	C3B^i^—C3B—C4B—C5B	−39.2 (8)
C3A^i^—C3A—C4A—C5A	−39.3 (9)	N1B—C3B—C4B—C5B	178.7 (11)
C3A—C4A—C5A—C5A^i^	40.4 (9)	C3B—C4B—C5B—C5B^i^	37.3 (8)

Symmetry codes: (i) *x*, −*y*+1/2, *z*.

### Hydrogen-bond geometry (Å, °)

**Table:**

*D*—H···*A*	*D*—H	H···*A*	*D*···*A*	*D*—H···*A*
N1A—H1A···S1A^ii^	0.86	2.53	3.367 (11)	166
N1B—H1B···S1B^iii^	0.86	2.76	3.483 (11)	142

Symmetry codes: (ii) −*x*+2, *y*−1/2, −*z*+2; (iii) −*x*+2, −*y*+1, −*z*+2.
